# Substance Use in Uninsured Cancer Survivors: A Multicenter Cross-Sectional Study of Free Clinics

**DOI:** 10.7759/cureus.10083

**Published:** 2020-08-27

**Authors:** Madeline MacDonald, Shreni Shah, Justin Swanson, Ethan Song, Tanzila Ahsan, Smitha Pabbathi, Rahul Mhaskar, Abu-Sayeef Mirza

**Affiliations:** 1 Internal Medicine, University of South Florida Morsani College of Medicine, Tampa, USA; 2 College of Public Health, University of South Florida, Tampa, USA; 3 Plastic Surgery, University of South Florida Morsani College of Medicine, Tampa, USA; 4 College of Arts and Sciences, University of South Florida, Tampa, USA; 5 Internal Medicine, Moffitt Cancer Center Department of Internal Medicine, Tampa, USA

**Keywords:** uninsured, free clinics, cancer, substance use, alcohol, tobacco

## Abstract

Introduction

Substance use disorders occur in about 5% of the cancer population and can decrease treatment adherence, impede pain management, and undermine a cancer survivor’s network of social support. Although current literature demonstrates substance use is associated with socioeconomic disparity, there is limited research on the prevalence of alcohol, tobacco, and illicit drug use among uninsured cancer survivors in the United States. Our multicenter cross-sectional study describes the prevalence of substance use in uninsured cancer survivors in the Tampa Bay Area.

Methods

A comprehensive retrospective chart review of electronic medical records and paper charts was conducted at nine free clinics in the Tampa Bay Area of Florida between January 1, 2016, and December 31, 2017. Substance use prevalence was compared between uninsured cancer survivors and uninsured patients without reported cancer history after adjusting for available demographic risk factors.

Results

There were 222 patients with a history of cancer and 6,768 patients without a history of cancer included for analysis. Cancer survivors had a median age of 55 years (interquartile range 48-61 years), were mostly female (n = 146, 66.1%), and of Hispanic ethnicity (n = 94, 52.5%). Cancer survivors were more likely to be current smokers (n = 42, 25.1%) compared to patients without a cancer history (n = 759, 16.1%). Patients with a history of cancer were more likely to be current drinkers (n = 34, 26%) compared to non-cancer patients (n = 942, 22.9%). There was no significant difference in illicit drug use history between the two groups.

Conclusions

Our study demonstrates that uninsured cancer survivors are more likely to be smokers and alcohol consumers than uninsured patients without a history of cancer. There was no significant difference in illicit drug use in cancer survivors and patients without a history of cancer. Future educational interventions should target substance use among uninsured cancer survivors.

## Introduction

Approximately 8% of Americans are diagnosed with alcohol abuse disorder; over 12% smoke cigarettes and roughly 33% use recreational drugs [1,2]. Tobacco use is the number one cause of preventable mortality in the United States, followed by obesity and then alcohol use [3]. Combined, alcohol and tobacco use leads to approximately 600,000 premature deaths each year in addition to causing disability, disease, and loss of productivity [3-5]. Studies have shown that substance use varies by race, ethnicity, household income, and insurance status [3]. Another factor contributing to substance use and abuse is psychological stress. Uninsured people suffer from higher rates of mental illness than those with insurance making them more susceptible to substance abuse [6]. The uninsured may be more likely to suffer the consequences of substance use due to barriers to receive medical care.

Alcohol, tobacco, and illicit drugs have numerous associations with morbidity and mortality, including several malignancies such as oropharyngeal, lung, and liver cancer [3,4]. The number of cancer survivors-defined as any person diagnosed with cancer has been steadily increasing in the United States over the past decade and is predicted to increase to 20 million people by 2026 [7]. Due to earlier detection and improved treatment modalities, both the incidence and death rate of cancer have decreased since 1999, leading to an increased number of survivors re-entering the medical system [7,8]. Survivors have unique health requirements that can differ from people without cancer and thus necessitate more specialized care; however, our current health system often does not provide a proper transition from oncology to primary care, leaving patients, particularly the poor and uninsured, without adequate resources [7].

Cancer survivors are a vulnerable population, especially when it comes to substance use, which can have serious negative consequences on their health [2,9,10]. While there are many studies that characterize substance use among the general population, relatively few describe substance use among cancer survivors. In addition, there is a paucity of research on uninsured cancer survivors. Substance use by cancer patients can not only undermine a patient’s social support system, but it can also decrease treatment adherence [2,9,10]. It is, therefore essential for healthcare providers to understand the patient’s risk of substance use and incorporate appropriate management into the treatment plan [10]. 

Although current literature demonstrates substance use is associated with socioeconomic disparity, there is limited research on the prevalence of alcohol, tobacco, and illicit drug use in uninsured cancer survivors in the United States. Our study describes the prevalence of substance use in uninsured cancer survivors in order to understand the unique needs of this community better.

## Materials and methods

This cross-sectional study includes uninsured patients served at nine free clinics in the Tampa Bay area in Florida between January 1, 2016, and December 31, 2017. Details of patient visits were aggregated across the study period to generate period prevalence data. Demographic and chronic disease parameters were collected via a retrospective review of paper and electronic medical records. Data abstraction was conducted by undergraduate and medical students from the University of South Florida, all of whom were trained on standardized abstraction procedures. Data were collected with an electronic data abstraction form and captured in REDCap [11]. 

Patients seen at any point during the study period were considered eligible for inclusion. Those without documentation discussing a positive or negative history of cancer were excluded from the analysis. Substance use histories were self-reported by patients seen in the free clinic setting. The format in which substance use history was recorded varied by the clinic but was typically recorded on patient intake forms or obtained as part of the social history. Prevalence of past or present tobacco smoking, alcohol consumption, or illicit drug use were collected and compared between cancer survivors - defined as a patient with any self-reported history of past diagnosis of cancer - and the comparison group being those patients denying a history of cancer. Present alcohol, tobacco, or illicit drug use was defined as any current use of alcohol, tobacco, or illicit drugs as documented in the chart at the time of the patient’s visit. Past use was defined as prior use with documentation of cessation. [12] Measures of association between cancer history and substance use are presented as crude odds ratios (OR) and adjusted odds ratios (aOR) after controlling for age (entered as a quadratic term after assessing linearity), sex, and race/ethnicity. Measures of association were generated using separate multiple logistic regression models for smoking, alcohol, and drug use. Goodness-of-fit was assessed for each model using the Hosmer-Lemeshow test. Missing demographic data were imputed using multivariate imputation by chained equations (MICE); neither substance use history nor cancer history were imputed and were thus analyzed on a complete case basis. Imputation of demographic data with MICE relies upon the assumption that these data were missing at random, while complete case analysis assumes that missing values were missing completely at random. All analyses were executed with R version 3.6.3 statistical software [13].

All participating clinics consented to the use of their data. This study was approved by the University of South Florida Institutional Review Board (Study # Pro00023920). 

## Results

During the two-year study period, 9,127 patients were served at the reviewed clinics. Of these, 6,990 (76.6%) had a documented positive or negative history of cancer; the remaining were excluded from the analysis. There were 222 (3.2%) patients with a history of cancer in our study sample. The most common cancers reported were breast (n = 50, 22.5%), prostate (n = 20, 9.0%), colon (n = 12, 5.4%), melanoma (n = 10, 4.5%), and lung (n = 9, 4.1%); this distribution was similar to previously reported findings using one year of data from this population [12]. Cancer survivors had a median age of 55 (interquartile range 48-61). The majority of cancer survivors were female (n = 146, 66.1%), unemployed (n = 78, 54.5%), and of Hispanic ethnicity (n = 94, 52.5%) (Table 1). 

**Table 1:** Demographic Characteristics of Patients Analyzed IQR = interquartile range *Missing values not included when calculating column percentages.

Characteristic	Cancer (N = 222)*	No Cancer (N = 6768)*
Age, median (IQR)	55 (48-61)	42 (26-54)
Sex		
Male	75 (33.9%)	2,756 (40.8%)
Female	146 (66.1%)	4,007 (59.2%)
Race/ethnicity		
Hispanic, all races	94 (52.5%)	2,752 (55.8%)
Caucasian	79 (44.1%)	1,282 (26.0%)
Black or African American	6 (3.4%)	696 (14.1%)
Asian	0 (0%)	153 (3.1%)
Other	0 (0%)	51 (1.0%)
Employment		
Employed	65 (45.5%)	1,932 (53.4%)
Unemployed	78 (54.5%)	1,685 (46.6%)

Of those patients with a recorded history of substance use, 42 (25.1%) cancer survivors were current smokers, and 36 (21.6%) were past smokers, while 759 (16.0%) and 470 (9.9%) of patients without a history of cancer were present or past smokers, respectively (Table 2). After adjusting for demographic confounders, cancer patients were more likely to report current tobacco smoking (aOR = 1.9, 95% CI [1.3, 2.7]) and past tobacco smoking (aOR = 1.8, 95% CI [1.2, 2.7]) (Table 2). Among cancer survivors, 33 (25.8%) were current consumers of alcohol, and 13 (10.2%) were past consumers; 940 (22.9%) patients without a history of cancer were current alcohol consumers, and 141 (3.4%) were past consumers. Of those patients with quantified current alcohol consumption, 5 (25%) cancer survivors and 66 (14.7%) patients without a history of cancer reported more than moderate consumption of alcohol (seven drinks or fewer for women and 14 or fewer drinks for men). Cancer survivors were more likely to report a history of alcohol consumption (aOR = 2.6, 95% CI [1.4, 4.8]). Cancer history trended towards a positive association with current alcohol consumption, although this was non-significant (aOR = 1.4, 95% CI [0.9, 2.1]). Adjusted associations between cancer history and current illicit drug use (aOR = 1.2, 95% CI [0.4, 3.3]) and past drug use (aOR = 0.8, 95% CI [0.4, 3.3]) were non-significant. The results of Hosmer-Lemeshow tests were non-significant for the models assessing the relationship between cancer history and smoking (χ² = 29.94, df = 24, p = 0.187), alcohol consumption (χ² = 32.49, df = 24, p = 0.115), or drug use (χ² = 23.00, df = 24, p = 0.520). 

**Table 2:** Prevalence of Substance Use and Measures of Association *Missing values not included when calculating column percentages. ^†^Adjusted odds ratios yielded from multiple logistic regression. Covariates included age, gender, race/ethnicity, and employment. The Hosmer-Lemeshow results were non-significant for models assessing smoking (χ² = 29.94, df = 24, p = 0.187), alcohol consumption (χ² = 32.49, df = 24, p = 0.115), or drug use (χ² = 23.00, df = 24, p = 0.520).

Substance Use	Cancer (N = 222)*	No Cancer (N = 6768)*	Crude OR [95% CI]	aOR [95% CI]^†^
Smoking status				
Never	89 (53.3%)	3509 (74.1%)	Reference group	Reference group
Past	36 (21.6%)	470 (9.9%)	3.0 [2.0, 4.5]	1.8 [1.2, 2.7]
Active	42 (25.1%)	759 (16.0%)	2.2 [1.5, 3.2]	1.9 [1.3, 2.7]
Alcohol consumption				
Never	82 (64.1%)	3026 (73.7%)	Reference group	Reference group
Past	13 (10.2%)	141 (3.4%)	3.4 [1.9, 6.3]	2.6 [1.4, 4.8]
Active	33 (25.8%)	940 (22.9%)	1.3 [0.9, 2.0]	1.4 [0.9, 2.1]
Drug use				
Never	106 (93.8%)	3524 (90.9%)	Reference group	Reference group
Past	3 (2.7%)	121 (3.1%)	0.8 [0.3, 2.6]	0.8 [0.2, 2.5]
Active	4 (3.5%)	232 (6.0%)	0.6 [0.2, 1.6]	1.2 [0.4, 3.3]

## Discussion

Our study reveals that uninsured cancer survivors seen in free clinics continue to be at risk for alcohol and tobacco use. Cancer survivors had a higher present and past history of tobacco smoking compared to the remainder of the free clinic population. While it is well understood that smoking is an independent risk factor for many cancers, according to several studies, it does not completely explain the increased incidence of cancer in low-income smokers [14]. Low income and uninsured individuals may have increased risk for occupational or environmental exposures to carcinogens, which may contribute to the increased cancer incidence in this population [14]. According to the Centers for Disease Control and Prevention, those with a GED level education, an annual household income less than $35,000, and those who are of uninsured status are significantly more likely to be smokers than those with higher education, higher income, and private health insurance [3]. Therefore, efforts should be made to provide uninsured patients with smoking cessation resources. 

Cancer survivors in the study were more likely to be current and past drinkers compared to the rest of the study population. Alcohol is also a known risk factor for several cancers, including oropharyngeal, laryngeal, esophageal, breast, liver, and colorectal cancer [15]. Several studies found that cancer risk may be reduced to that seen in never drinkers after greater than 20 years of alcohol cessation [15]. Another study found that the risk of cancer-specific mortality is increased significantly in moderate drinkers and heavy drinkers [16]. Thus, uninsured individuals may be at increased risk for alcohol abuse and its complications [17]. 

There was no significant difference between current or past illicit drug use in cancer survivors and the larger clinic population. As compared to the general population, substance abuse disorders reportedly only occur within 5% of the cancer population [9]. However, the low incidence of substance abuse in cancer patients might be due to underreporting, as one study showed that 28% of cancer patients abuse alcohol, and between 14% and 58% use tobacco [1,18]. For uninsured patients, in particular, there are very few resources that aid with substance abuse management [10,14]. There are over 1000 free clinics in the United States that provide care for over 1.8 million uninsured patients; however, less than 10% of these clinics offer substance use services such as alcohol counseling or tobacco cessation counseling [6,19]. 

Although they do not detract from our conclusions, our study has several limitations. Data collection relied on accurate reporting by patients seen at free clinics and precise and complete documentation by clinicians. Some patients may have failed to disclose or may have underreported their substance use history or pertinent past medical history. Besides age, demographic and substance use information was collected inconsistently. This study was underpowered to meaningfully detect differences in substance use between cancer types. While this study included a sizable sample from nine free clinics in Tampa, our population’s ethnic and racial heterogeneity may not be generalizable to other communities. 

## Conclusions

Our study found that uninsured cancer survivors are more likely to drink alcohol and smoke tobacco but are not significantly more likely to use illicit substances when compared to uninsured patients without a history of cancer. Our findings suggest that uninsured cancer survivors continue to be at risk for tobacco and alcohol use. Unfortunately, many patients with polysubstance use are of a lower socioeconomic status and may not seek out appropriate care due to stigma or lack of resources to access healthcare. More research needs to be done to evaluate substance use among uninsured cancer survivors, and efforts need to be made to refer this vulnerable population of patients to appropriate resources. By highlighting the substance use patterns in this population, we hope free clinics may provide better healthcare support for this vulnerable population.
